# Predictors of Reducing Sexual and Reproductive Risk Behaviors Based on the Information-Motivation-Behavioral Skills (IMB) Model among Unmarried Rural-To-Urban Female Migrants in Shanghai, China

**DOI:** 10.1371/journal.pone.0062787

**Published:** 2013-04-30

**Authors:** Yong Cai, Ying Wang, Zhijie Zheng, Jin Wang, Wen Yao, Jin Ma

**Affiliations:** 1 School of Public Health affiliated with School of Medicine, Shanghai Jiaotong University, Shanghai, PR China; 2 Shanghai Pudong Population and Family Planning Management Center, Shanghai, PR China; 3 Shanghai Hongkou Center for Disease Control and Prevention, Shanghai, PR China; Tehran University of Medical Sciences, Islamic Republic of Iran

## Abstract

**Background:**

Due to the increase of premarital sex and the lack of reproductive health services, unmarried rural-to-urban female migrants experience more risks of sex and reproductive health (SRH). This study was designed to describe SRH related knowledge, attitude and risk behaviors among unmarried rural-to-urban female migrants and examine the predictors of reducing sexual and reproductive risk behaviors based on information-motivation-behavioral skills (IMB) model and to describe the relationships between the constructs.

**Methods:**

We conducted a cross-sectional study to assess SRH related information, motivation, behavioral skills and preventive behaviors among unmarried rural-to-urban female migrants in Shanghai, one of the largest importers of migrant laborers in China. Structural equation modeling (SEM) was used to assess the IMB model.

**Results:**

A total of 944 subjects completed their questionnaires. The mean age was 21.2 years old (SD = 2.3; range 16 to 28). Over one-fourth of participants reported having had premarital sex (N = 261, 27.6%) and among whom 15.3% reported having had the experience of unintended pregnancy, 14.6% with the experience of abortion. The final IMB model provided acceptable fit to the data (CFI = 0.99, RMSEA = 0.034). Reducing sexual and reproductive risk behaviors was significantly predicted by SRH related information (β = 0.681, *P*<0.001) and behavioral skills(β = 0.239, *P*<0.001). Motivation (β = 0.479, *P*<0.001) was the significant indirect predictor of reducing sexual and reproductive risk behaviors mediated through behavioral skills.

**Conclusions:**

The results highlight the importance and necessity of conducting reproductive health promotion among unmarried rural-to-urban female migrants in China. The IMB model could be used to predict reducing sexual and reproductive risk behaviors and it suggests future interventions should focus on improving SRH related information and behavioral skills.

## Introduction

Migration is a global health issue and challenge for public health all around the world. Due to the rapid urbanization in the coming decades, more and more rural migrant workers move across counties or even provinces to make monetary gains through employment [1]. China’s economic success story in the last three decades cannot be separated from its plentiful supply of migrant labor from the rural areas in the hinterland to cities along the coast [2]. However, migrants usually face more health risks because of the restrictive barriers of household registration system in the cities of China [3]. Migrant workers have been identified as a population at risk for sex and reproductive health (SRH) such as unplanned pregnancies, sexual transmitted infections (STI) and human immunodeficiency virus infection/acquired immunodeficiency syndrome (HIV/AIDS) in many counties [4], [5], [6], [7]. It is estimated that there were approximately 230 million rural-to-urban migrants in China in 2011 with the average age of 28 years old, 49.6% of which were female [8]. In China, most migrants moved from western regions to metropolises such as Shanghai, Beijing and Guangzhou, etc. For example, Shanghai holds a population of about 20 million and over 1 in 3 were migrants in 2011 [9]. Recently, many studies had focused on the risky behaviors related to HIV/AIDS among male migrants or migrant female sex workers of China [10], [11], [12]. However, few studies engaged in general rural-to-urban female migrants with special physiological and psychological characteristics who maybe much more vulnerable to risky health behavior than males as they are newly exposed to risk factors in the urban environment [13], [14].

In China, there were more married than single males but more single females than married females among the migratory population [13]. Among rural-to-urban female migrants, unmarried individuals were dropped out of the reproductive health services from the family planning policy of China. Meanwhile, the sexual attitude became more and more open and the premarital sexual behaviors among youth increased quickly in the past decades [14], [15]. The rapid changes in sexual mores also impacted women’s opinions such as the increasing freedom of sexual expression, and control over their bodies and sexual choices [16]. Studies have shown that instances of premarital sexual behaviors among unmarried female migrants ranged from 17–80%, depending on their job functions in China [14], [17], [18], [19]. Unmarried young female migrants exposed themselves to SRH risk such as unintended pregnancies, induced abortion, venereal infections and STI/AIDS [19], [20]. It is suggested that the reproductive health promotion for unmarried young females in China, including this migrant population, needs to be conducted without delay [20].

To our knowledge, there are few studies aimed at sexual and reproductive risk behaviors using health promotion theoretical frameworks in China [21], and no studies focused on unmarried rural-to-urban female migrants based on the theoretical frameworks such as the information-motivation-behavioral skills (IMB) model. The IMB model developed by Fisher and his colleagues held that HIV prevention information, motivation, and behavioral skills are the fundamental determinants of HIV preventive behavior [22]. This model has been tested of good fitness in many studies of STI/AIDS preventions among risk population recently [23], [24], [25]. The IMB model assumed that an individual must be well-informed, motivated and possess the self-efficacy behavioral skills to decreases risk behaviors. The constructs of the IMB model and the relationships among them had been well-supported across HIV risk populations and health promotion behaviors, however, little work had been done in SRH among female migrants.

This study will describe the SRH related knowledge, attitude and risk behaviors among unmarried rural-to-urban female migrants. Assumed SRH prevention information and motivation affect preventive behavior (reducing sexual and reproductive risk behaviors) largely through behavioral skills and information, and motivation may also have direct effects on it, we also aimed to test the associations among IMB constructs as predictors of sexual and reproductive risk behaviors by using structural equation modeling (SEM) in unmarried rural-to-urban female migrants of Shanghai, China.

## Methods

### Study Site

Shanghai is one of the largest metropolitan areas in China with a population over 23 million, among whom 39% is itinerant population. Recently, the number of female migrants has increased rapidly recently, with over 30% over the age of 15 being single. The first HIV case in Shanghai was reported in 1987, while by the end of 2011, 7498 HIV cases had been cumulatively reported. Over 65% new HIV cases were migrants reported in 2010, among whom HIV/AIDS transmission was largely attributed to heterosexual contact [26].

### Study Population and Sampling Size

Between June and August 2012, we recruited unmarried female migrants using the cluster sample method. Three districts (Changning, Pudong and Hongkou) of Shanghai were randomly selected firstly. The medicine center provided physical examination for migrant workers of every district chosen. Individuals were eligible if they were unmarried female migrants aged over 16 years.

Assuming a prevalence of the premarital sexual behaviors of 30.0% [14], an α of 0.05, and a relative error of sampling of 0.15P, we calculated a required sample size of 1000 to allow for the larger sampling error of cluster sample method and an non-response rate of 10%. Of the 1000 migrants who volunteered to participate in the study, 979 (97.9%) were eligible for participation and of those, 944(96.4%) finished the field survey effectively. Each participant was paid the equivalent of 20 RMB (US$ 3.17) for study participation.

### Ethics

The study was reviewed and approved by the National Nature Science Fund Committee. It was also approved by the Ethics Committee of School of Public, Shanghai Jiaotong University. All participants were given written informed consent including the objectives and the procedure of the study, as well as potential risks and benefits of participating in the study before enrollment.

### Data Collection

Researchers from the School of Public Health of Shanghai Jiaotong University surveyed the participants using interview-led structured questionnaires which had been tested in preliminary research and showed suitability by the reliability and validity analysis [27]. Prior to participation, we explained the aim and major content of the survey to each interviewee and emphasized that participation was voluntary and anonymous. The procedure lasted approximately 20 minutes and was conducted face-to-face in a private room. The questionnaires included information about demographics, such as age, education, hometown, living status, monthly income, and occupation, and the constructs of the IMB model, which included SRH prevention information, motivation, behavioral skills, and sexual and reproductive risk behaviors.

### Measures

The latent variables included information, motivation and behavioral skills which were hypothesized to reflect key constructs of the IMB model [25]. Preventive behaviors were used as the main outcome and the dependent variable in the model. Each latent variable was constructed with several observable variables which can be observed and directly measured. Each measure of the IMB model construct is described below.

#### Information

SRH related information was measured with 22 items with “yes”, “no” or “do not know” to assess their knowledge about reproductive health and STI/AIDS. Positive answers were credited with a score of one, while negative answers or responses of “do not know” received a score of zero. One indicator containing eight items was related to reproductive health knowledge (RHK; e.g. Do you think the most risk period of conception is about 3 days before menstrual bleeding?; Do you think it can prevent pregnancy by oral contraceptive?). Factor analysis showed the equal coefficients for each of the eight items, suggesting that the sum of these items would be suitable composite scale. It was converted into a total score as RHK (Cronbach’s alpha coefficient = 0.79; range of 0–8). The other indicator containing fourteen items was related to STI/AIDS knowledge (SAK; e.g., Do you think people can catch AIDS by sexual behavior?; Do you think syphilis can be transmitted to infant from positive mother?). Factor analysis suggested that the sum of these fourteen items would be a suitable composite scale. It was converted into a total score as the scale of STI/AIDS (Cronbach’s alpha coefficient = 0.74; range of 0–14). The higher the scores, the more information with SHR knowledge the participants caught.

#### Motivation

The motivation to practice a SRH preventive act was assumed to be a function of one’s attitudes or relevant subjective norms toward the SRH. It was measured by two indexes constructed from answers on a 5-point Likert scale (1 completely agree to 5 completely disagree). One was the permissive attitude towards sex including 4 items (PAS; e.g. Do you agree a girl to have premarital sex with her boyfriend; Do you agree with premarital cohabitation). The sum of each item’s score was converted into a total score as the scale of a permissive sex attitude (Cronbach’s alpha coefficient = 0.82; range of 4–20). The other was the attitude to reproductive health, which contained 5 items (e.g. Do you agree with premarital pregnancy; Do you agree with frequent abortion). The sum of each item’s score was converted into a total score as the scale of the attitude toward reproductive health (ARH; (Cronbach’s alpha coefficient = 0.71; range of 5–25). The lower the score, the more permissive attitude to SRH the participant held.

#### Behavioral skills

Respondents’ perceived self-efficacy to perform key SRH preventive skills was assessed using two scales. One scale was self-efficacy to safe sex and consisted of 4 items (e.g. Do you think you can use condom during your sexual debut?; Do you think you can discus safe sex with your partner?) All the 4 items constructed from answers on a 5-point Liket scale (1 completely unable to 5 completely able). The sum of the 4 item’s score was converted into a total score as the scale of the self-efficacy to safe sex (SESS; Cronbach’s alpha coefficient = 0.72; range of 4–20). The other scale was the self-efficacy to RH containing 4 items (e.g. Have you ever wanted to get the health promotion of RH?; Do you have the effective way for RH consultation?). The sum of the 4 items’ scores with the answers on a 4-point Liket scale (1 definitely no to 4 definitely yes) was changed into a total score as the scale of the self-efficacy to RH (SERH; Cronbach’s alpha coefficient = 0.69; range of 4–16). The higher the score, the more self-efficacy to SRH preventive skill the participant got.

#### Preventive behaviors

The reduction of risk behaviors could also act as the preventive behavior [22], [25] Literature reviews showed no established standard of sexual and reproductive risk behaviors measures associated with the IMB model [21], [22], [25]. We constructed sexual and reproductive risk behaviors with 6 items (e.g. Do you have the experience of premarital sex; Do you have the experience of unintended pregnancy?; Do you have the experience of abortion; Do you have the experience of STI; Do you have the experience of venereal infections; Do you not use condom during sexual intercourse?) which had been tested with good reliability and validity in the previous study [27]. Each item was measured with “yes” (credited with a score of zero) or “no” (received a score of one). The sum of the 6 item’s scores was converted into a total score as the scale of the prevention behaviors (Reducing **s**exual and reproductive risk behaviors; Cronbach’s alpha coefficient = 0.76; range of 0–6).The higher the score, the less sexual and reproductive risk behaviors the participant experienced. The score of sexual and reproductive risk behaviors was used as the main outcome and the dependent variable in this study.

### Statistical Analysis

Descriptive statistics, such as means, standard deviations, frequencies, and percentages were reported to describe the characteristics of our study population using SPSS 20.0. The hypothetical IMB was examined by the structural equation model (SEM) using the Amose20.0. Model fit was examined by using the comparative fit index (CFI), the root mean square error of approximation (RMSEA), and the maximum likelihood chi-square values/degrees of freedom ratio, etc [28]. A CFI value greater than 0.9 and a RMSEA value lower than 0.05 indicating a good fit of the model [29]. A non-significant likelihood ratio chi-square test suggests the well model fit, however, chi-square is sensitive to sample size, therefore, with a 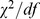 ratio of 3 or less indicating acceptable fit [28]–[29]. Confirmatory factor analysis (CFA) was conducted to examine the factor structure (measurement model) and the relationships among all the latent variables and manifest variables [21]. A path model was performed to examine the predicting factors of SRH based IMB model.

## Results

### Characteristic of Participants

A total of 944 unmarried female migrants completed all measure of the questionnaire (Table 1). Participants were, on average, 21.2 years old (SD = 2.3; range: 16 to 28), and most came from Midwest China (N = 768, 81.4%). Most participants completed junior or senior high school (N = 679, 71.9%) and 70.4% earned a monthly income from 1500–3000 Chinese Yuan (1 USD = 6.34 CNY). A large proportion of participants were found to be working in the service industries (N = 596, 63.1%), and others were working in the manufacturing enterprises.

**Table 1:** Socioeconomic, demographic characteristics and sexual and reproductive risk behaviors of the participants.

Characteristic variables	N	%
Age (Years)		
16–20**	311	32.9
21–27	633	67.1
Hometown		
Midwest	176	18.6
East	768	81.4
Education		
Junior school	371	39.3
Senior school	308	32.6
College	265	26.1
Money income (CRMB*)		
<1500	68	7.2
1500–3000	665	70.4
>3000	211	22.4
Occupation		
The service industries	348	36.9
The manufacturing enterprises	596	63.1
Experience of premarital sex		
Yes	261	27.6
No	683	72.4
Experience of unintended pregnancy		
Yes	40	15.3
No	221	85.7
Experience of abortion		
Yes	38	14.6
No	223	85.4
Experience of STI		
Yes	4	1.5
No	257	98.5
Experience of venereal infections		
Yes	78	29.9
No	183	60.1
Condom use		
Yes	139	53.3
No	122	46.7

* CRMB: Chinese Yuan; 6.34CNY = 1 USD.

** In China, 20 years is youngest legal age for marriage among female.

Over one-fourth of participants reported having had premarital sex (N = 261, 27.6%), among whom 15.3% reported having had the experience of unintended pregnancy, 14.6% with the experience of abortion, 29.9% with the experience of venereal infections. About half of the participants with experience of sexual intercourse reported consistent condom use (N = 139, 53.3%).

### Confirmatory Factor Analysis

We constructed a preliminary confirmatory factor analysis (CFA) model to estimate the factor structure and relationships among all of the IMB variables. The means, standard deviations, ranges, and factor loadings for all scales of the IMB model were shown in the Table 2. All factor loadings were significant in the model (p<0.05). Knowledge about RH was low; the average knowledge test score was 4.9 out of a possible 8 correct responses. The knowledge about STI/AIDS was even lower than RH; the mean score was 7.41 out of a possible 14 correct responses.

**Table 2:** Summary statistics and factor loadings of the IMB model in confirmatory factor analyses.

Scales	Range	Mean	SD	FL*
Information				
Reproductive health knowledge (RHK)	0–8	4.90	1.97	0.71
STI/AIDS knowledge (SAK)	0–14	7.41	2.87	0.73
Motivation				
Permissive attitude to sex(PAS)	4–20	15.01	2.85	0.88
Attitude toward reproductive health(ARH)	5–25	22.55	2.48	0.94
Behavior Skill				
Self-efficacy to safe sex(SESS)	4–20	14.81	2.56	0.82
Self-efficacy to reproductivehealth(SERH)	4–16	12.71	2.05	0.76
Sexual and reproductive risk behaviors	0–6	5.43	1.02	0.88

* FL = factor loadings, all significant<0.001.

**Figure 1:**
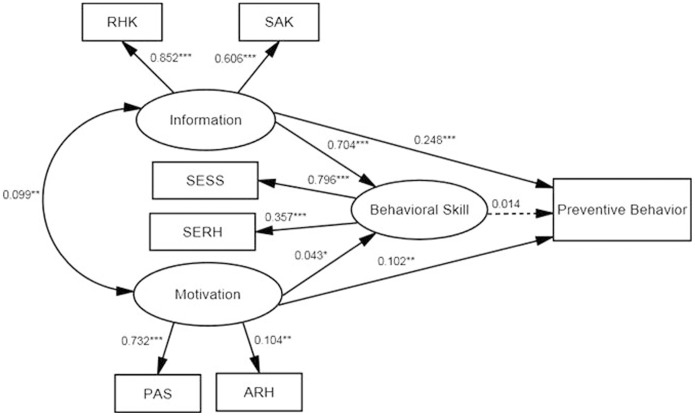
The preliminary IMB model predicting preventive behavior among 944 unmarried rural-to-urban female migrants. Oval represent multiple-indicator latent variables, rectangle represent single-indicator observable variables. Single-headed arrow represent regression path, double-headed arrows represent correlations. Regression coefficients are standardized (* p<0.05, ** p<0.01, *** p<0.001). Dotted line indicates non-significant path from original IMB model.

The initial IMB model appeared in Figure 1. However, the model fit statistics for the preliminary model were not acceptable: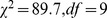, and the 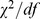 ratio was not within the acceptable range of 3 or less (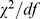 = 9.9). The value of CFI was acceptable 0.917, but the value of RMSEA was not within the acceptable range of 0.05 or less (RWSEA = 0.097). Overall, it indicated the initial model needed to be modified.

### Path Model of IMB

**Figure 2:**
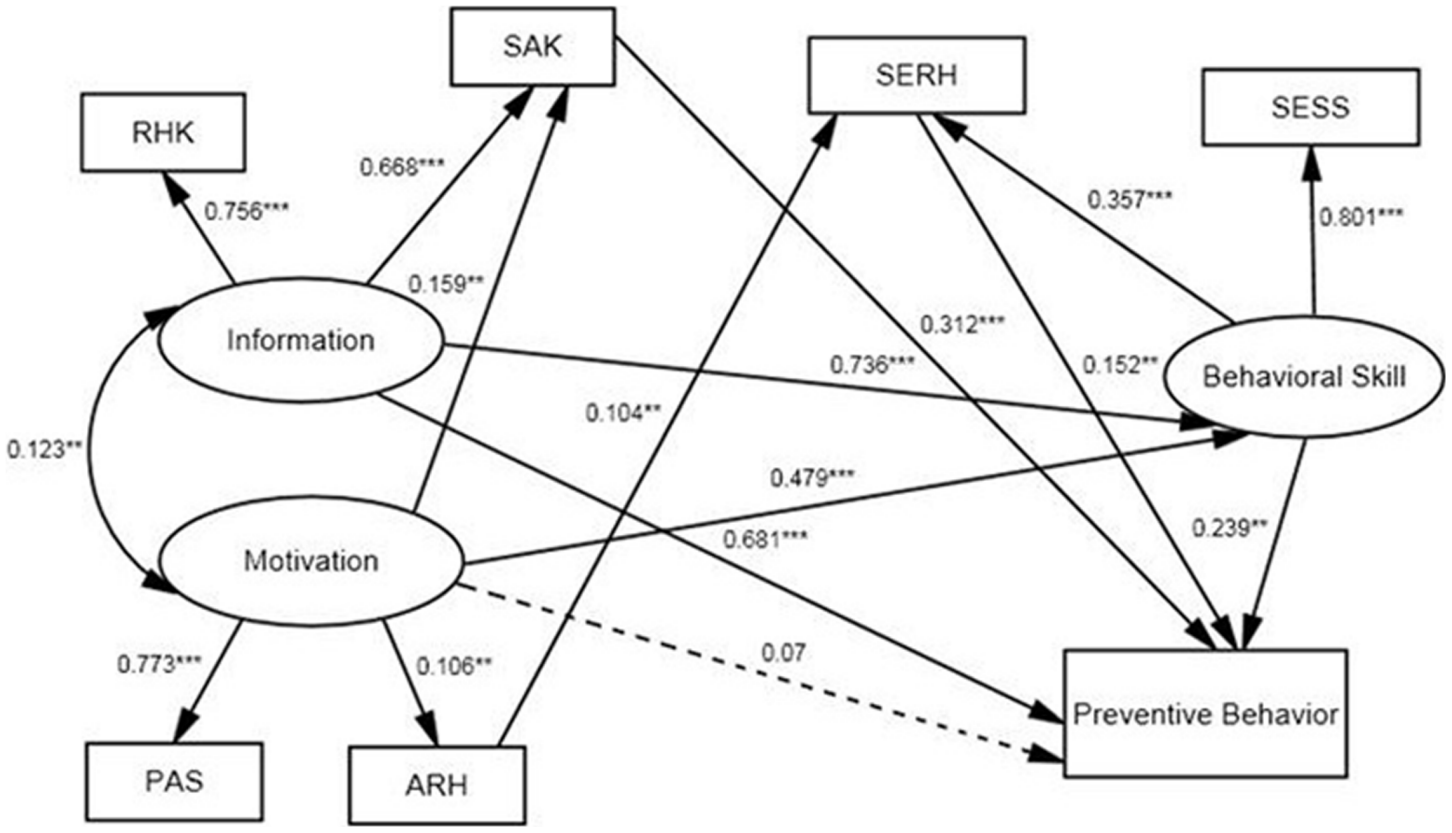
The final IMB model predicting preventive behavior among 944 unmarried rural-to-urban female migrants. Oval represent multiple-indicator latent variables, rectangle represent single-indicator observable variables. Single-headed arrow represent regression path, double-headed arrows represent correlations. Regression coefficients are standardized (** p<0.01, *** p<0.001). Dotted line indicates non-significant path from original IMB.

We modified the IMB model by adding supplementary paths to the initial model.The final IMB model is depicted in Figure 2. And the fit indices for the model were acceptable after modification: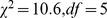, P = 0.061, the 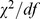 ratio = 2.1, CFI = 0.99, RMSEA = 0.034. It indicated a well-fitting structure of the final IMB model.

As expected, we found sexual and reproductive risk behaviors was strongly predicted by behavior skill (β = 0.239, *P*<0.001) and information (β = 0.681, *P*<0.001), while motivation (β = 0.07, *P>*0.05) was not significantly associated with reducing sexual and reproductive risk behaviors. Motivation (β = 0.479, *P*<0.001) and information (β = 0.736, *P*<0.001) were significantly and positively associated with behavior skill. Otherwise, four new paths were indicated in the final model. It showed the SAK was not only a part of information but also related to motivation (β = 0.159, *P*<0.05). The knowledge about STI/AIDS was also a motivation to practice a SRH preventive act. As a result, the SAK (β = 0.312, *P*<0.001) strongly and positively predicted reducing sexual and reproductive risk behaviors. The new path between ARH and SERH showed that attitude might directly determinate self-efficacy to reproductive health (β = 0.104, *P*<0.01), which also positively affect reducing sexual and reproductive risk behaviors (β = 0.152, *P*<0.01).

## Discussion

Unmarried adolescents often suffer from sexual and reproductive health problems due to their pre-marital sexual practices. For women aged 15 to 19, complications of pregnancy, childbirth, and unsafe abortion are the major causes of death [30]. In China, due to limited SRH services, unmarried rural-to-urban female migrants are usually facing much more risk of STI/AIDS, unintended pregnancy, and other health consequences than their urban peers. The result of this research indicates that nearly 28% of unmarried rural-to-urban female migrants report having had premarital sex, which is similar to the finding among three major cities in China in 2011 [14]. In relation to RH and STI/AIDS knowledge, as compared to studies conducted in their urban peers using the similar questionnaire [31], [32], our results showed that the SRH related information among unmarried rural-to-urban female migrants are poorer, especially the information about STI/AIDS. The results suggest an urgent need for STI/AIDS and RH related interventions for the unmarried rural-to-urban female migrants.

Our study also focuses on the applicability of the IMB model in predicting sex and reproductive preventive behaviors among this population. The IMB model represents the first attempt to specify the specific linkages among constructs related to HIV/AIDS prevention. It posits that individuals will maintain HIV/AIDS preventive behavior if they are well-informed, are motivated to prevent HIV/AIDS, and perceive themselves as possessing the behavioral skills to act successfully [33]. Assuming SRH prevention information and motivation also affect prevention behaviors by behavioral skills and information and motivation may also have direct effects on SRH behaviors, we construct the original IMB model among unmarried rural-to-urban female migrants in our research. Consistent with the original IMB model, having more information (higher level of RH and STI/AIDS knowledge), having more personal motivation (positive attitude toward reproductive health and permissive attitude to sex), and having more preventive skills (higher self-efficacy to safe sex and reproductive health) was associated with the SRH prevention behaviors.

It indicates the original IMB model is not acceptable and it seems the behavioral skills are not associated with preventive behavior directly. Although current study findings show that the behavioral skills component might not be significantly associated with preventive behavior in the IMB model [25], [34], we suggest the original IMB model be modified by path analysis. In the final model with well-fitting structure, we find that not only information but also the behavioral skills are significantly associated with the preventive behaviors. Many researchers have suggested that information is an important but unnecessary precursor to HIV-risk prevention behavior among higher HIV/AIDS risk population [21], [22]. But to our knowledge, information will have a direct association with behavior when both are measured at the same level of specificity (e.g. SRH information and preventive behaviors). As the RH and STI/AIDS knowledge levels are very low among unmarried rural-to-urban female migrants, it is important and necessary to popularize related information among them. It also suggests enhancing self–efficacy behaviors to safe sex and reproductive health will have direct effects during reproductive health intervention among unmarried rural-to-urban female migrants. We find there is no significant association between motivation and preventive behavior in the final model, however, motivation contributes to preventive behavior indirectly by affecting information and behavioral skills. The results suggest that the future intervention should strengthen the understanding about reproductive health information and behavioral skills to reduce risk behaviors among unmarried rural-to-urban female migrants.

Our study has several limitations. First, it is self-report data from the participants, who might misreport their behaviors or attitudes because sex remains a sensitive topic in China. While this bias cannot be determined from the data, reliability and validity studies had indicated good test-retest and good fit of the results for the questionnaire we used. Second, the results of our study were based on cross-sectional data which limited utility for causality analysis. We test a theoretical model by using structural equation model with specified paths and measure the constructs from cross-sectional data, which can only examine the associations between constructs observed at a single point in time, not causally. Third, the direction of the effects can not be determined from the cross-sectional data. Longitudinal data is needed to observe the effects of information, motivation and behavioral skills on changes in sexual and reproductive risk behaviors in the future research. Thus, it will provide a more comprehensive understanding of the elements that should be incorporated in future interventions of SRH among unmarried rural-to-urban female migrants.

Nevertheless, our study is the first known investigation of the utility of the IMB model for predicting reducing sexual and reproductive risk behaviors among unmarried rural-to-urban female migrants. The findings highlight the importance and necessity of conducting reproductive health promotion among them. The IMB model may offer an effective theoretical foundation for reducing sexual and reproductive risk behaviors and it suggests that interventions among unmarried rural-to-urban female migrants should focus on improving SRH related information and behavioral skills.
